# Stable Marital Histories Predict Happiness and Health Across Educational Groups

**DOI:** 10.1007/s10680-025-09733-x

**Published:** 2025-05-13

**Authors:** Miika Mäki, Anna Erika Hägglund, Anna Rotkirch, Sangita Kulathinal, Mikko Myrskylä

**Affiliations:** 1 Population Research Institute at the Family Federation of Finland, Helsinki, Finland; 2 Center for Social Data Science, University of Helsinki, Helsinki, Finland; 3 University of Turku, Turku, Finland; 4 Department of Mathematics and Statistics, University of Helsinki, Helsinki, Finland; 5 Max Planck Institute for Demographic Research, Rostock, Germany; 6 Max Planck – University of Helsinki Center for Social Inequalities in Population Health, Helsinki, Finland; 7 Helsinki Institute for Demography and Population Health, University of Helsinki, Helsinki, Finland

**Keywords:** Partnership history, Resource substitution, Cumulative disadvantage, Health, Quality of life

## Abstract

Couple relations are a key determinant of mental and physical well-being in old age. However, we do not know how the advantages and disadvantages associated with partnership histories vary between socioeconomic groups. We create relationship history typologies for the cohorts 1945-1957 using the Survey of Health, Ageing, and Retirement in Europe and examine, for the first time, how relationship histories relate to multiple indicators of well-being by educational attainment. The results show that stable marriages predict greater well-being, compared to single and less stable partnership histories. The positive outcomes are similar across all educational groups. Those with lower education who have divorced experience even lower well-being in old age. The interaction analyses suggest that individuals with fewer resources could suffer more from losing a partner. The findings underscore that current and past romantic relationships are linked to well-being in old age and help policymakers identify vulnerable subgroups among the ageing population.

## Introduction

Romantic couple relations are among the most intimate, lasting and important relationships in our lives and a cornerstone of emotional, social, and economic well-being (Wängqvist et al., 2016; Luyckx et al., 2014). Previous research has exhaustively shown that having a partner is positively associated with a multitude of well-being outcomes in later life, such as life satisfaction, quality of life, health, morbidity, and mortality (Wong & Waite, 2015; Han et al., 2014; Holt-Lunstad et al., 2008; Coombs, 1991; Manzoli et al., 2007; Waite, 1995; Wang et al., 2022; Reneflot & Mamelund, 2012). Indeed, having a spouse is arguably one of the single most important factors for healthy ageing (Wood et al., 2009). At the same time, the proportions of single, divorced, or remarried elderly have increased (Cherlin, 2017), and relationship biographies have become more diverse. It is crucial to identify what types of partnership histories are related to increased vulnerability, and to what extent lacking or losing a partner can be compensated for.

Having a partner as such may not improve well-being. Instead, the association between partnerships and well-being could emerge from the partnership history as the social, economic and health advantages of partnerships accumulate over time (cf. DiPrete & Eirich, 2006). Existing literature, however, tends to examine relationships either as single events, such as marriage or divorce, or depict them rather statically through the current partnership status (Sassler, 2010; Schütz, 2019; Gumà et al., 2014). So far, a few studies have conceptualised partnerships as trajectories (Zimmermann & Hameister, 2019; Roberson et al., 2018; Jung, 2023; O’flaherty et al., 2016). These suggest that aspects of partner histories predict well-being beyond the current status, and that further insights into well-being can be gained by forming trajectories (Peters & Liefbroer, 1997; Jung, 2023; Zimmermann & Hameister, 2019). A first contribution of this paper is that we conceptualise relationships as trajectories and see whether they are linked with well-being.

Several scholars, including Becker (1973), have stressed that the gains from marriage are likely to depend on individual traits, such as attractiveness, intelligence, and education. These different gains may arise because of homogamy, higher marital satisfaction, and more fulfilling family life and ties. So far, scholars have not considered education in greater detail. Instead, educational attainment has been treated as a control rather than a stratifying feature (Boyce et al., 2016; Han et al., 2014; Wong & Waite, 2015). To our knowledge, only one study in recent decades has examined whether the association between marital status and well-being indicators varies by education: Øien-Ødegaard et al. (2021) found that low education increased the suicide risk among separated.

There are several reasons to assume that the gains from a stable partnership history follow an educational gradient. On the one hand, highly educated individuals might profit more from having a lifelong partner than those with a lower education due to lower conflict (Woszidlo & Segrin, 2013), more fulfilling family life (Roberts & Dunbar 2011; Margolis & Myrskylä 2011) and the possible compounding effect of different types of resources that both education and a stable relationship provide (Zajacova & Lawrence, 2018; Wood et al., 2009). This process would imply resource multiplication. On the other hand, those with a lower educational level may rely more heavily on a spouse, as stable partnerships compensate for a lack of other resources. Put differently, spousal loss might have more detrimental consequences for those with fewer resources (DiPrete & Eirich, 2006). The latter process, in turn, suggests resource substitution.

In this study, we analyse two such dimensions that have not been studied jointly before: whether particular relationship histories predict well-being and whether the well-being associations of those histories vary by education. We investigate whether romantic relationship histories are associated with well-being for men and women and how the associations differ between educational groups of Northern and Western Europeans in third age, a stage in one’s life between middle age and old age (Gilleard & Higgs, 2007). Specifically, we are interested in signs of resource multiplication or substitution: is the combination of having a certain educational level and relationship trajectory linked with even higher or lower well-being outcomes?

Empirically, we create partnership history typologies with sequence analysis using retrospective life history interview data from the Survey of Health, Ageing, and Retirement in Europe (SHARE) from 13 European countries. We measure well-being with subjective health and life satisfaction and conduct robustness checks with grip strength and CASP-12 quality-of-life indicator. Our analyses control for several confounding measures including childhood conditions, income, and number of children. The results are further corroborated by a confounder analysis.

### Why are Relationship Status and Well-Being Linked?

Empirical evidence for the interconnectedness of marital status and well-being is conclusive. Current marital status and the quality of couple relationships are generally linked to well-being (see review in Wood et al., 2009). These associations seem to prevail in different societies: Diener et al. (2000) studied the relationship between marital status and subjective well-being in 42 countries and found that differences in effect sizes are negligible. Verbakel (2012) concludes with a similar study design that "normative climate appears to hardly affect well-being gaps between partnership statuses". Thus, we expect partnerships to support well-being similarly across our study population. But what explains the consistency of these findings? Previous research points to three mechanisms, namely selection into marriage, marriage as a key social role, and the everyday social support of living with a spouse (Dush & Amato, 2005; Averett et al., 2013).

First, marriage itself might not make people happy and healthy. Instead, happy and healthy people could self-select into marriage (Koball et al., 2010). Although some selection effects are at play (see Goldman, 1993, for a theoretical overview), the majority of longitudinal studies suggest that selection alone does not account for all the positive effects of relationships on well-being (Horwitz et al., 1996; Wood et al., 2009; Fu & Noguchi, 2018; Grover & Helliwell, 2019). Yet not all agree (Mastekaasa, 1992; Ludwig & Brüderl, 2018), highlighting that results may vary with methodological design.

Second, the structural symbolic interactionist perspective argues that roles with a high level of commitment result in high levels of identity and self-worth (Stryker & Statham 1985, as cited in Dush & Amato 2005). Internalising the role of a wife or husband (or parent) shapes both the behaviour and self-perception, and consequently enhances well-being, although this depends on their commitment to the given identities (Stryker, 1959). The fact that few want to live without a long-term romantic partner even in wealthy, individualised, and liberal societies (Kontula 2016; Rotkirch 2020, pp. 40-41) demonstrates the high value attached to couple relations.

Third, marriage tends to provide social support and regulation beyond any other form of relationship (Ross, 1995; Coombs, 1991; Scott, 2000). In high-income societies, unions are the primary household unit for bread-winning, consumption, and intimacy. Particularly in the ageing population, the typical alternative to living with a romantic partner is to live alone (Rindfuss & Vandenheuvel, 2022; Becker, 1991; Rotkirch, 2020). Individuals who are attached to social networks are healthier and live longer (House et al., 1988); those with a spouse have someone in the same household to share the joys and sorrows of life in the long term, which single adults often lack.

### Relationships and Well-Being from a Life Course Perspective

Well-being in old age reflects combined experiences over the entire life course, not just the present moment. Studying sequences of relationship statutes, or relationship histories, could reveal dynamics that are concealed in analyses focusing on relationship statuses. The focus on sequences aligns with life course theory: Individual outcomes are the result of previous trajectories and events. These evolve with time, are embedded in social structures, and form patterns of social stratification (Elder et al., 2003; Diewald & Mayer, 2009).

Empirical research points to the value of analysing relationship trajectories. For instance, marriage dissolution has long-term, negative implications on well-being and health, which persist even among those who remarry (Hughes & Waite, 2009). Correspondingly, unstable partnerships, multiple relationship transitions, and long-term singlehood are associated with higher levels of depression and stress and lower social and emotional support (Jung, 2023; Zimmermann & Hameister, 2019). Partnership trajectories characterised by stable unions typically display the highest levels of psychological and physical well-being (O’flaherty et al., 2016; Tambellini et al., 2024). The association between partnership histories and well-being is likely to increase with age, since inequalities in health, resources, and subjective well-being tend to accumulate as individuals grow older (Kratz & Patzina, 2020; Ross & Wu, 1996; Prus, 2007; Headey, 2010). Finding a new partner, however, can at least partly compensate for more unstable partnership trajectories (Jung, 2023).

Overall, this process aligns with the framework of cumulative advantage/disadvantage, suggesting that negative or positive implications in one aspect of life increase over time (Dannefer, 2003). As relationship statuses are associated with lifestyles, health behaviour, social support, and networks, well-being—or lack thereof—is likely to spill over to other domains and accumulate as individuals age. Melo et al. (2019) goes as far as claiming that the ageing process as such is a result of accumulated dis/advantages over the years from different spheres of life.

In this study, we use cumulative dis/advantage to refer to the compound well-being effects of relationship trajectories over time. Additionally, we hypothesise how educational attainment could potentially amplify or mitigate cumulative dis/advantage (see Sect. 1.4).

### Gender Differences

Empirical research highlights that the implications of romantic relationships differ by sex. In particular, men seem to benefit significantly from being in a partnership (Coombs, 1991; Metsä-Simola & Martikainen, 2013); long-term singlehood substantially lowers men’s well-being (Zimmermann & Hameister, 2019; Jung, 2023).

For women, by contrast, the association between partnerships and well-being appears weaker (O’flaherty et al., 2016; Zimmermann & Hameister, 2019). At first glance, this seems counterintuitive, as women face greater economic disadvantages following union dissolution than men (see Mortelmans, 2020, for a review). For instance, women’s salary growth rates decline more sharply, leaving them at a long-term economic disadvantage (Raz-Yurovich, 2013). Additionally, women are more likely to take on primary childcare responsibilities after a breakup, which compounds their financial strain.

Despite these economic disadvantages, several factors could explain why relationship breakups tend to have a greater detrimental effect on men’s well-being, particularly in later life. Women are more likely to engage in behaviours that promote health and often influence their partners to do the same (Reczek et al., 2016; Umberson, 1992; Westmaas et al., 2002; Lewis & Butterfield, 2007). Men, on the other hand, tend to have smaller and less emotionally supportive social networks in later life (Ajrouch et al., 2005). While single mothers bear a heavier financial burden after union dissolution, they often maintain stronger bonds with their children and receive more care in old age. Fathers who do not live with their children after a breakup may instead pay a higher social price. (Mortelmans, 2020)

Furthermore, women often experience greater psychological distress from staying in marriages with low relationship quality (Bulanda et al., 2016; Brown & Wright, 2017; Carr & Utz, 2020). This may make them more inclined than men to end an unsatisfactory relationship and better equipped to cope with life without a significant other (Rosenfeld, 2018).

### Educational Resource Multiplication or Substitution

Stable unions are likely to increase well-being, while unstable histories and long-term singlehood lower it. However, the outcomes of relationship histories might follow an educational gradient. Education anchors the accumulation of human, social, and personal capital (O’Rand, 2001), and is positively associated with a multitude of well-being outcomes (see Zajacova & Lawrence, 2018, for review). Theoretically, it remains unclear whether high- or low-educated benefit more from stable unions. In the following, we argue that two mutually exclusive patterns are possible: *resource multiplication* and *resource substitution*.

On the one hand, high education might strengthen the positive association of stable marital histories on well-being. Stable relationships allow for the accumulation of resources, and highly educated have more resources to sustain long-term relationships to begin with: they are likely to partner with similarly positioned individuals (Schartz, 2010), receive more support from the kin network (Roberts & Dunbar, 2011), and are better equipped to deal with conflict (Woszidlo & Segrin, 2013). These factors contribute to longer and happier unions among highly educated (Tavakol et al., 2017). Stable unions, in turn, provide a platform for resource accumulation.

Highly educated men and women could also profit more from marital stability. For example, combined financial resources (Jung, 2023), spousal emotional support, fulfilling relationships with kin-network (Roberts & Dunbar, 2011), or the joys of raising children could be more pronounced among highly educated individuals (Margolis & Myrskylä, 2011). Likewise, since both education and stable marriages predict health and life satisfaction (Zajacova & Lawrence, 2018; Wood et al., 2009), their interplay could result in multiplicative well-being outcomes. Thus, we assume resource multiplication if high education coupled with stable partnership histories mutually reinforce well-being (Ross & Mirowsky, 2006).

Alternatively, low-educated individuals might profit more from stable partnership trajectories than highly educated. As low-educated individuals generally have fewer resources (Ross & Mirowsky, 2010), their well-being might rely more on stable partnership histories. The loss of spousal financial and emotional support is likely to influence well-being more strongly in this group than among the highly educated, who can buffer the negative consequences of unstable trajectories and living alone with other social, economic, and psychological resources (Ferraro et al., 2009). This pattern would suggest resource substitution (Mirowsky & Ross, 2005): stable union histories would have stronger benefits, or conversely, single or fragmented union histories stronger disadvantages among low-educated individuals, as they have fewer resources of resilience.

Theoretically, resource substitution and multiplication should not co-exist: either the benefits of a stable partnership history are stronger for those with higher education (resource multiplication), the benefits of a stable partnership history are stronger for those with lower education (resource substitution), or there are no educational interaction effects (cf. Ross & Mirowsky, 2006).

### Hypotheses

Based on previous research, we formulate a set of hypotheses. First, following cumulative advantage and disadvantage, we assume that stable marital relationship histories are associated with higher well-being after age 60 (1a), while histories characterised by instability or long-term singlehood are associated with lower well-being (1b). In addition, we expect (1c) more robust and consistent results for men, but possibly also some associations for women.

We anticipate that the link between partnership histories and well-being could vary by education, but the direction is not clear. Given the mutual exclusivity of resource substitution and multiplication, and the lack of conclusive findings in previous research, we remain agnostic as to which, if either, will be supported by our study. If the educational gradient is in line with resource multiplication, we expect high education to predict even stronger positive associations between stable partnership histories and well-being (2a). Education would then amplify the cumulative advantage of having a stable marital history as the highly educated profit more from family life. If resource substitution occurs, we expect that low education predicts stronger negative well-being outcomes for lifelong single and unstable partnership histories (2b). In such case, the low educated could neither utilise educational nor spousal resources, compared to those with high education—who can buffer unstable partnership trajectories with resources attached to education.

## Data and Methods

Life histories were generated with the help of the SHARELIFE interviews of the Survey of Health, Ageing, and Retirement in Europe (Börsch-Supan & Bergmann, 2019).^1^ SHARE is a micro-panel data infrastructure that covers households with at least one member over 50 years of age in all EU countries, Switzerland, and Israel. The survey collects both panel and retrospective life course data. The retrospective SHARELIFE pseudo-panel interviews were conducted in 2008 and 2017 (wave 3 and 7, respectively). Respondents were asked to report, amongst others, on their childhood circumstances as well as their past partners, including all cohabitational, marital, and dating relationships. If the life history interviews were conducted in 2008, any changes in relationship statuses were updated with subsequent panel interview data.

Our analyses are based on Northern and Western Europe as defined by United Nations (UNSD, 1999). The sample was restricted to these geographical areas to ensure that our study population would come from roughly similar cultures in terms of family formation and family life patterns (Klüsener, 2015). As a robustness check, we examined whether patterns varied between welfare states and cultures within Northern and Western Europe by stratifying the inferential analyses for welfare regimes following the classification scheme in Eikemo et al. (2008): Nordic (Sweden, Denmark, Finland), Baltic (Estonia, Latvia, Lithuania), and Bismarckian (Austria, Belgium, France, Germany, Luxembourg, Netherlands, Switzerland) welfare regimes.

We selected respondents born between 1945 and 1957 for two reasons: they were at least 60 years old in 2017 when the data were collected and were all part of the baby boomer generation (Bavel & Reher, 2013). This generation formed unions at a time when the prevalence of marriage was at its peak. Yet, this is also a generation when premarital cohabitation and non-marital childbearing started to become more common, starting from Northern Europe. (Perelli-Harris & Amos, 2015; Klüsener, 2015). The final sample size for analysis was 18,256 individuals. In order to obtain an adequate sample size, all couples were heterosexual.

Here, we categorise partnership statuses into unmarried, first and higher-order marriages, dating, cohabitation, divorce, and widowhood. This takes differences between partnered and unpartnered individuals more exhaustively into consideration (Næss et al., 2021; Pinquart, 2003): Although cohabitation and marriage increasingly resemble each other both legally and socially (Cherlin, 2020; Perelli-Harris & Amos, 2015), there is still a clear difference in terms of commitment, longevity, and symbolic importance (Barlow et al., 2001; Cherlin, 2004; Rault, 2019; Brown & Wright, 2017). Similarly, first- and higher-order marriages differ in terms of stability, relationship satisfaction, and demographic makeup (Zahl-Olsen et al., 2019; Hägglund et al., 2021; Booth & Edwards, 1992). Thus, later life outcomes could differ as well. We also have information on dating relationships. In SHARELIFE, dating is defined as a romantic relationship in which couples do not live at the same address most of the time. Dating here includes those who have a romantic relationship before moving in together, as well as those who permanently are in a living apart together (LAT) relationship (Lewin, 2017). Such relationships tend to be more flexible and less committed than marriage or cohabitation (Régnier-Loilier, 2016; Duncan & Phillips, 2010). In our operationalisation, an individual is dating if they enter a romantic relationship without co-residence, irrespective of previous status.

In a first step, we create relationship history typologies. By partnership history, we mean the ordered series of relationship statuses from adolescence to old age (cf. Cornwell, 2015, p. 21).^2^ The partnership trajectories were created by sequence analysis or agglomerative hierarchical clustering (see Cornwell, 2015, for a theoretical overview). We first created a distance matrix with the dynamic hamming method. The substitution costs of two states at position *p* are calculated as follows:

1 $$\begin{aligned} s_{p} (A,B) & = 4 - \left( {pr(X_{p} = A|X_{{p - 1}} = B) + pr(X_{p} = B|X_{{p - 1}} = A)} \right. \\ & \quad \left. { + pr(X_{{p + 1}} = A|X_{p} = B) + pr(X_{{p + 1}} = B|X_{p} = A)} \right) \\ \end{aligned}$$

where $s_p(A,B)$ is the substitution cost at position^3^*p* and $X_p$ is the state^4^ at the $p^{th}$ position. The method assesses both the probability of being in state *A* and state *B* at position *p* as well as the transition probabilities from *A* to *B* at position *p* and vice versa. This means that both timing and order are taken into account. (Cornwell, 2015, p.128) Thus the substitution costs are calculated automatically based on how common a certain transition is at a given age. For example, a transition from marriage to widowhood will be given a much higher cost at the age of 20 than 60. We chose to use dynamic hamming method due to the fact that the substitution costs are derived empirically from the data. We still found very similar clusters with dynamic hamming and different variants of optimal matching.

The distance matrix between the individual trajectories was further analysed using Ward’s agglomerative clustering method to form clusters (Ward, 1963). The clusters are obtained by minimising the within-cluster sum of squares, and hence produce groups with similar histories. We also experimented with other algorithms but the clustering alternatives with Ward’s method made the most sense conceptually. It is calculated as follows:

2 $$\begin{aligned} \begin{aligned} \Delta (J, K)&=\sum _{i \in J \cup K}\left\| \vec {x}_{i}-\vec {m}_{J \cup K}\right\| ^{2}-\sum _{i \in J}\left\| \vec {x}_{i}-\vec {m}_{J}\right\| ^{2}-\sum _{i \in K}\left\| \vec {x}_{i}-\vec {m}_{K}\right\| ^{2} \\&=\frac{n_{J} n_{K}}{n_{J}+n_{K}}\left\| \vec {m}_{J}-\vec {m}_{K}\right\| ^{2}, \end{aligned} \end{aligned}$$

where $\Delta$ is the merging cost of arbitrary clusters *J* and *K*, and $\vec {m}_{J}$,$\vec {m}_{K}$ are the centres of individual and combined clusters, and $\vec {x}_{i}$ is an individual in a given cluster. If there are *n* clusters at a given point of the process, $$\left( {\begin{array}{c}n\\ 2\end{array}}\right)$$ distances are calculated and the clusters with the smallest distance—or within-cluster sum of squares or *d* are merged. (Kaufman & Rousseeuw, 1990, p. 231)

We performed goodness-of-fit tests to decide how many clusters to choose: average silhouette width (ASW) indicates high between-group dissimilarity and high within-group similarity; Hubert’s Gamma (HG) measures the capacity of the clusters to reproduce the distances (Studer, 2013). In addition, we corroborated the test statistics by inspecting the clustering trees visually.

Initially, we added a second sequence channel with offspring histories, but the clusters depended almost entirely on the completed number of children—regardless of which algorithm we used. Since we do not focus on the associations between childbearing and parenting here, we dropped the children channel and added the number of children as a control variable for some of our models.

The typologies were created jointly for men and women for two reasons. First, we wanted to compare differences in well-being outcomes by sex, and having the same typologies for both groups made the comparison easier. Second, the relationship histories of heterosexual men and women are interrelated by definition.

Health was measured by a subjective assessment^5^ and well-being by self-reported life satisfaction,^6^ as they are direct and established ways to estimate well-being (Fischer, 2009; Jenkinson, 2013).

Robustness checks were carried out with grip strength—an objective estimator of health that predicts advantageous active ageing (Bohannon, 2019)—and CASP-12, an indicator capturing the quality-of-life in old age with robust theoretical underpinnings^7^ (Borrat-Besson et al., 2015). All well-being outcomes were measured at the respondent’s most recent interview, but no later than in 2017 (wave 7).

We treated subjective health and life satisfaction as continuous rather than ordinal to preserve comparability with Grip Strength and CASP-12. For the same reason, we standardised all continuous and ordinal variables. As Agresti (2015, pp. 214-215) points out, using OLS for ordinal response variables can lead to misleading results because of ceiling and floor effects. Consequently, we performed robustness checks with ordinal regression to ensure that such effects were not present.

We analysed the associations between partnership histories and later life outcomes with six linear regression models. The first model had relationship clusters as independent variables, but only controlled for age. In the second model, control variables that were exogenous to the clusters themselves were added to model 1: family composition at the age of 10 years, and childhood socioeconomic background measured by the number of books, features, and rooms at home, and whether the respondent experienced hunger as a minor, all asked retrospectively (see Havari and Mazzonna (2015) for a discussion about the reliability of these measures). The third model was an over-controlled one: it made sure that the cluster associations were not merely artefacts of the number of children, highest educational attainment, or household income at the time of interview. Naming it ’over-controlling’ stems from the fact that these control variables are at least in part shaped by the relationship histories themselves, and in any case occur after the start of the trajectories. For models 4, 5, and 6, we added interactions between educational attainment and the clusters. Otherwise, they were identical to models 1, 2, and 3, respectively.

Missing values (please refer to Tables 1, 2) were handled with multiple imputation with 6 imputed datasets. Adult information did not predict childhood variables and cluster membership did not predict any other variables. Otherwise, we used all variables that were used in the analyses.

To further analyse the robustness of the associations, we examined how an unmeasured confounder would alter our results for the first three models. To this end, we computed an E-value (VanderWeele & Ding, 2017) that approximates how strong an unmeasured confounder associated with both the independent and dependent variable, conditional on the covariates, would have to be in order to explain away their relationship. It does not make any assumptions about the confounder. However, it is no more conservative than other often used sensitivity analyses and is straightforward to implement. (Ding & VanderWeele, 2016)

E-value is calculated as:

3 $$\begin{aligned} \begin{aligned} E-value&= RR + \sqrt{RR \cdot (RR - 1)}&if\,RR\ge 1 \\&=RR' + \sqrt{RR' \cdot (RR' - 1)}&if\,RR<1 \end{aligned} \end{aligned}$$

where *RR* stands for risk ratio and $RR'$ its inverse 1/*RR*. Before that, the estimates of linear regression are transformed to the risk ratio scale. The transformation takes into account effect sizes and standard errors:

4 $$\begin{aligned} \begin{aligned} RR&\approx exp\{0.91 \cdot d\} \\ CI&\approx exp\{0.91 \cdot d \pm 1.78 \cdot se\} \end{aligned} \end{aligned}$$

where *d* is the standardised effect size and *se* the standard error of *d* (VanderWeele & Ding, 2017). The approximation relies on assumptions from meta-analyses to convert standardised effect sizes into odds ratios and further the obtained odds ratios into risk ratios (VanderWeele, 2017; Hasselblad & Hedges, 1995; Borenstein et al., 2021). Note that by definition, the E-value equals one if the upper and lower confidence bounds of the corresponding risk ratios are under and above one, respectively. This becomes evident when we look at the definition in Eq. 3: if the confidence bounds of the E-values crossed one, the interpretation of the upper/lower tail would become meaningless.

All analyses were conducted with the Puhti supercomputer R singularity container (CSC, 2021; R Core Team, 2021): Sequence analyses and clustering trees with Graphviz and the R package TraMineR (Gabadinho et al., 2011; Gansner & North, 2000); all other visualisations with ggplot2 and jtools (Wickham, 2016; Long, 2020); multiple imputations with mice (van Buuren & Groothuis-Oudshoorn, 2011); unmeasured confounding with EValue (VanderWeele & Ding, 2017); interaction forecasting with emmeans (Lenth, 2021), and tables with Table 1 and stargazer (Rich, 2021; Hlavac, 2018).

## Results

Taken together, our analyses identified five typologies of relationship histories, demonstrated that stable marital trajectories were associated with the highest well-being across all educational groups, and finally, that low education combined with trajectories characterised by divorce without repartnering displayed even lower well-being associations.

Table 1 depicts the descriptives of the regression covariates and an overview of the prevalence of relationship statuses between ages 15 and 60. The higher educated tend to have better well-being in later life and are more attached to romantic relationships.

**Table 1:** Descriptive statistics by gender and education

	Male			Female		
	Higher	Secondary	Basic	Higher	Secondary	Basic
	(N=3224)	(N=3188)	(N=1694)	(N=3900)	(N=3686)	(N=2337)
**Subjective health**						
Mean (SD)	3.1 (1.1)	2.8 (1.0)	2.7 (1.0)	3.0 (1.0)	2.8 (1.0)	2.7 (1.0)
Missing	10 (0.3%)	11 (0.3%)	4 (0.2%)	5 (0.1%)	4 (0.1%)	3 (0.1%)
**Life satisfaction**						
Mean (SD)	8.1 (1.5)	7.8 (1.7)	7.6 (1.8)	7.9 (1.6)	7.7 (1.8)	7.7 (1.8)
Missing	68 (2.1%)	96 (3.0%)	53 (3.1%)	54 (1.4%)	49 (1.3%)	44 (1.9%)
**Grip strength**						
Mean (SD)	45.6 (8.4)	45.2 (8.6)	43.9 (9.0)	28.4 (5.7)	27.7 (6.0)	26.8 (5.9)
Missing	45 (1.4%)	39 (1.2%)	26 (1.5%)	51 (1.3%)	45 (1.2%)	41 (1.8%)
**CASP-12**						
Mean (SD)	40.0 (5.3)	39.2 (5.8)	37.9 (6.2)	39.5 (5.6)	38.7 (6.0)	38.0 (6.5)
Missing	44 (1.4%)	47 (1.5%)	42 (2.5%)	61 (1.6%)	38 (1.0%)	30 (1.3%)
**No. of rooms (aet. 10)**						
Mean (SD)	4.3 (2.5)	3.9 (1.9)	3.9 (1.7)	4.1 (2.2)	3.8 (1.8)	3.9 (1.8)
Missing	148 (4.6%)	147 (4.6%)	61 (3.6%)	208 (5.3%)	141 (3.8%)	77 (3.3%)
**No. of features at home (aet. 10)**						
Mean (SD)	3.1 (1.9)	2.5 (1.9)	2.1 (1.8)	2.9 (1.9)	2.5 (1.9)	2.1 (1.7)
Missing	14 (0.4%)	13 (0.4%)	10 (0.6%)	9 (0.2%)	7 (0.2%)	4 (0.2%)
**No. of books at home (aet. 10)**						
None or very few	431 (13.4 %)	922 (28.9 %)	761 (44.9 %)	453 (11.6 %)	960 (26.0 %)	1043 (44.6 %)
One shelf (11-25 books)	685 (21.2 %)	920 (28.9 %)	422 (24.9 %)	771 (19.8 %)	1060 (28.8 %)	632 (27.0 %)
One bookcase (26-100 books)	1126 (34.9 %)	867 (27.2 %)	353 (20.8 %)	1437 (36.8 %)	1076 (29.2 %)	437 (18.7 %)
Two bookcases (101-200 books)	437 (13.6 %)	229 (7.2 %)	80 (4.7 %)	570 (14.6 %)	300 (8.1 %)	106 (4.5 %)
More than two bookcases	523 (16.2 %)	198 (6.2 %)	50 (3.0 %)	647 (16.6 %)	239 (6.5 %)	79 (3.4 %)
Missing	22 (0.7%)	52 (1.6%)	28 (1.7%)	22 (0.6%)	51 (1.4%)	40 (1.7%)
**Living with parents (aet. 10)**						
Yes	2907 (90.2 %)	2769 (86.9 %)	1417 (83.6 %)	3402 (87.2 %)	3158 (85.7 %)	2005 (85.8 %)
No	298 (9.2 %)	390 (12.2 %)	260 (15.3 %)	463 (11.9 %)	511 (13.9 %)	313 (13.4 %)
Missing	19 (0.6%)	29 (0.9%)	17 (1.0%)	35 (0.9%)	17 (0.5%)	19 (0.8%)
**Hunger in childhood**						
No	3144 (97.5 %)	3084 (96.7 %)	1637 (96.6 %)	3838 (98.4 %)	3607 (97.9 %)	2274 (97.3 %)
Yes	38 (1.2 %)	44 (1.4 %)	31 (1.8 %)	41 (1.1 %)	57 (1.5 %)	49 (2.1 %)
Missing	42 (1.3%)	60 (1.9%)	26 (1.5%)	21 (0.5%)	22 (0.6%)	14 (0.6%)
**Number of children**						
2+	2298 (71.3 %)	2151 (67.5 %)	1114 (65.8 %)	2759 (70.7 %)	2562 (69.5 %)	1700 (72.7 %)
1	497 (15.4 %)	580 (18.2 %)	301 (17.8 %)	711 (18.2 %)	781 (21.2 %)	426 (18.2 %)
0	429 (13.3 %)	457 (14.3 %)	279 (16.5 %)	430 (11.0 %)	343 (9.3 %)	211 (9.0 %)
**Ever married**						
Yes	2986 (92.6 %)	2900 (91.0 %)	1471 (86.8 %)	3555 (91.2 %)	3452 (93.7 %)	2182 (93.4 %)
No	238 (7.4 %)	288 (9.0 %)	223 (13.2 %)	345 (8.8 %)	234 (6.3 %)	155 (6.6 %)
**Ever divorced**						
Yes	485 (15.0 %)	494 (15.5 %)	260 (15.3 %)	759 (19.5 %)	728 (19.8 %)	394 (16.9 %)
No	2739 (85.0 %)	2694 (84.5 %)	1434 (84.7 %)	3141 (80.5 %)	2958 (80.2 %)	1943 (83.1 %)
**Ever cohabited**						
Yes	1507 (46.7 %)	1396 (43.8 %)	686 (40.5 %)	1792 (45.9 %)	1467 (39.8 %)	794 (34.0 %)
No	1717 (53.3 %)	1792 (56.2 %)	1008 (59.5 %)	2108 (54.1 %)	2219 (60.2 %)	1543 (66.0 %)

### Partnership Typologies

**Fig. 1:**
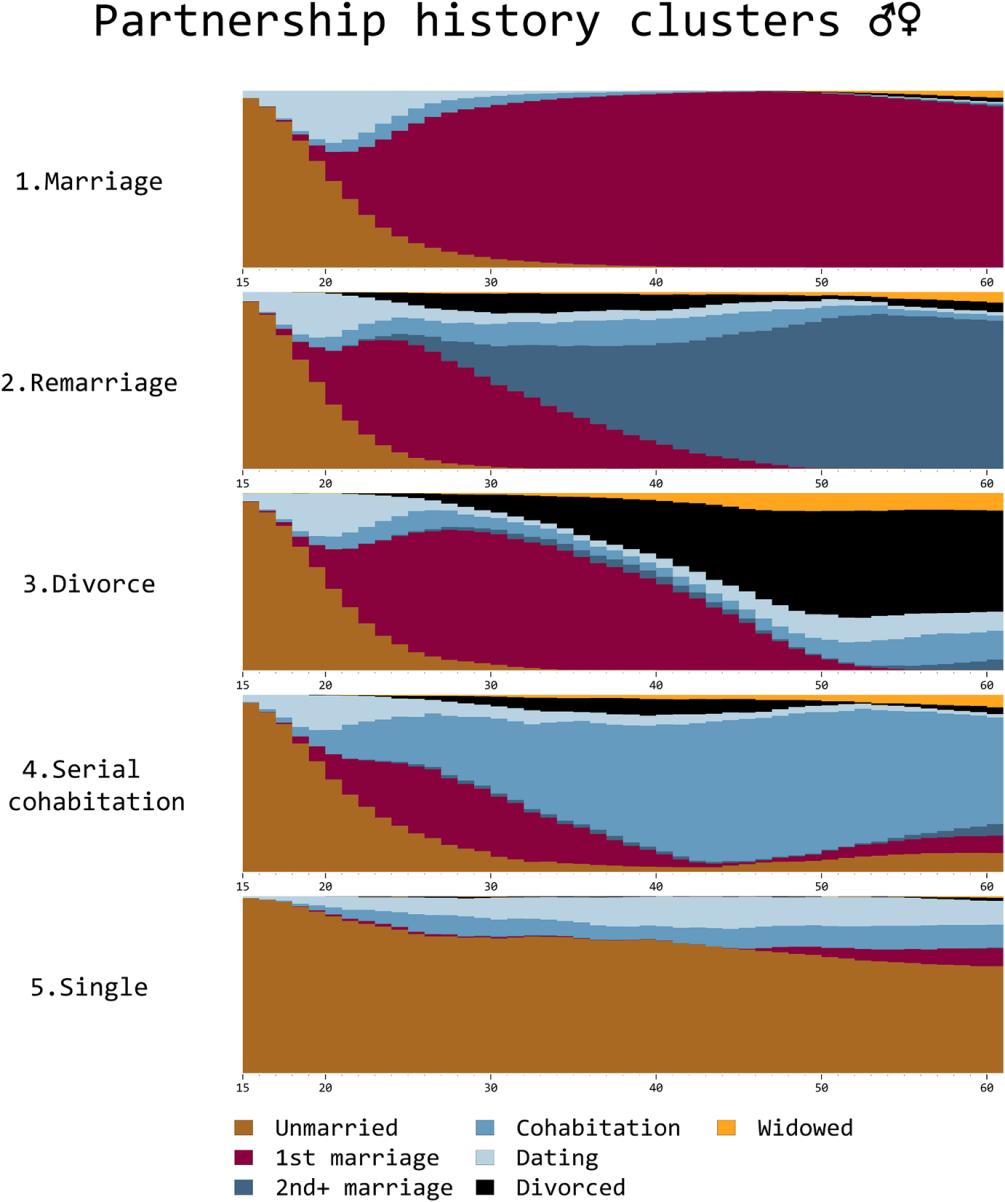
Partnership history clusters for Western and Northern Europeans born in 1945–1957 at the age of 15–60

We start by describing the relationship trajectory typologies. We chose a five-cluster solution (Figs. 1 and 9) based on visual inspection and goodness-of-fit tests (Figs. 5 and 6). The *marriage* (1) cluster is characterised by a brief period of dating—and sometimes cohabitation—followed by a permanent first marriage, usually starting during their 20s. More than half of our study population belongs to this group. The *remarriage* (2) cluster tended to marry around their 20s and divorce within the first ten years of marriage. Most would remarry in their 30s. The higher-order marriages were often preceded by cohabitation. The *divorce* (3) cluster is identical to the first two clusters until early 30s. However, unlike in the remarriage cluster (2), people remained single or widowed, or started dating or cohabiting after divorce, but did not generally remarry. In the *serial cohabitation* (4) cluster, cohabiting and dating were prominent throughout the life course. Yet, there was a considerable amount of variation in the relationship stages after the initial period of singlehood. Some were married between ages 20-40, but they usually did not stay divorced for long before starting to date or cohabit (see Fig. 9). The sequence index plot (Fig. 9) demonstrates that this cluster was, in fact, predominantly characterised by repeated as opposed to stable cohabitation. Finally, the vast majority in the *single* (5) cluster never co-resided and many of them never dated, either. Those who did, generally returned to singlehood in a few years, or had several short relationships (see Fig. 9 for individual life courses).

**Table 2:** Descriptive statistics by cluster

	1.Marriage	2.Remarriage	3.Divorce	4.Serial cohabitation	5.Single	Overall
	(N=12135)	(N=1960)	(N=1720)	(N=1189)	(N=1206)	(N=18210)
**Year of birth**						
Mean (SD)	1950.6 (3.6)	1950.7 (3.6)	1951.0 (3.6)	1951.4 (3.6)	1951.0 (3.7)	1950.8 (3.6)
**Gender**						
Male	5610 (46.2 %)	865 (44.1 %)	550 (32.0 %)	532 (44.7 %)	646 (53.6 %)	8203 (45.0 %)
Female	6525 (53.8 %)	1095 (55.9 %)	1170 (68.0 %)	657 (55.3 %)	560 (46.4 %)	10007 (55.0 %)
**Education**						
Higher	4768 (39.3 %)	756 (38.6 %)	664 (38.6 %)	461 (38.8 %)	475 (39.4 %)	7124 (39.1 %)
Secondary	4555 (37.5 %)	812 (41.4 %)	659 (38.3 %)	433 (36.4 %)	415 (34.4 %)	6874 (37.7 %)
Basic	2690 (22.2 %)	383 (19.5 %)	381 (22.2 %)	283 (23.8 %)	294 (24.4 %)	4031 (22.1 %)
Missing	122 (1.0%)	9 (0.5%)	16 (0.9%)	12 (1.0%)	22 (1.8%)	181 (1.0%)
**Income**						
Mean (SD)	2676.5 (14396.5)	2603.4 (15796.4)	2496.8 (7764.3)	2941.1 (9428.9)	2418.8 (5347.4)	2651.3 (13337.5)
Missing	775 (6.4%)	87 (4.4%)	61 (3.5%)	60 (5.0%)	54 (4.5%)	1037 (5.7%)
**Country**						
Austria	992 (8.2 %)	149 (7.6 %)	165 (9.6 %)	86 (7.2 %)	105 (8.7 %)	1497 (8.2 %)
Belgium	1642 (13.5 %)	231 (11.8 %)	271 (15.8 %)	163 (13.7 %)	144 (11.9 %)	2451 (13.5 %)
Denmark	1084 (8.9 %)	235 (12.0 %)	113 (6.6 %)	154 (13.0 %)	86 (7.1 %)	1672 (9.2 %)
Estonia	1273 (10.5 %)	283 (14.4 %)	201 (11.7 %)	167 (14.0 %)	127 (10.5 %)	2051 (11.3 %)
Finland	607 (5.0 %)	71 (3.6 %)	72 (4.2 %)	80 (6.7 %)	66 (5.5 %)	896 (4.9 %)
France	1277 (10.5 %)	156 (8.0 %)	186 (10.8 %)	117 (9.8 %)	144 (11.9 %)	1880 (10.3 %)
Germany	1363 (11.2 %)	257 (13.1 %)	121 (7.0 %)	61 (5.1 %)	105 (8.7 %)	1907 (10.5 %)
Latvia	405 (3.3 %)	84 (4.3 %)	90 (5.2 %)	31 (2.6 %)	31 (2.6 %)	641 (3.5 %)
Lithuania	533 (4.4 %)	68 (3.5 %)	92 (5.3 %)	28 (2.4 %)	30 (2.5 %)	751 (4.1 %)
Luxembourg	479 (3.9 %)	48 (2.4 %)	46 (2.7 %)	12 (1.0 %)	37 (3.1 %)	622 (3.4 %)
Netherlands	647 (5.3 %)	68 (3.5 %)	60 (3.5 %)	29 (2.4 %)	76 (6.3 %)	880 (4.8 %)
Sweden	1012 (8.3 %)	175 (8.9 %)	166 (9.7 %)	189 (15.9 %)	134 (11.1 %)	1676 (9.2 %)
Switzerland	821 (6.8 %)	135 (6.9 %)	137 (8.0 %)	72 (6.1 %)	121 (10.0 %)	1286 (7.1 %)

Overall, the clusters resemble each other in demographic characteristics (see Table 2), although there are a few exceptions: men were over-represented in the singlehood (5) and women in the divorce (3) cluster. While educational differences were generally insubstantial, men with stable relationship trajectories tended to be more educated. For women, the opposite pattern prevailed (see Fig. 7). The distribution of relationship clusters did not vary much across countries. One stable marital trajectories were by far the most common, and single or serial cohabitation trajectories were the rarest. Further inspection by multinomial regression revealed that Northern European respondents were more likely to belong to the serial cohabitation cluster. No other distinct patterns were observed (see Table 3 and Fig. 8).

### Well-being Outcomes

**Fig. 2:**
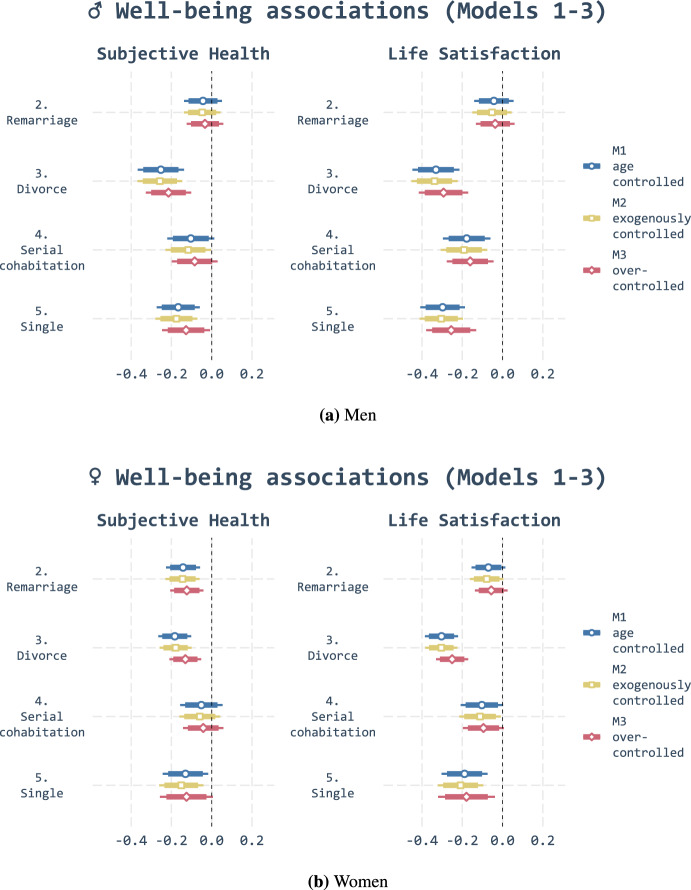
Linear regression for subjective health and life satisfaction without interaction effects (Models 1,2 & 3). *Note:* Marriage (c1) is the reference category

Next, we analyse the association between partnership histories and two measurements of well-being, namely subjective health and life satisfaction. The results for main effects (models 1-3) are depicted in Fig. 2, where the marriage (1) cluster is our reference category. As all non-binary variables were standardised, an effect size of one would imply a difference of one standard deviation in the response variable compared with the reference category. The white circles and squares represent point estimates, while the thick and narrow bars the 95% and 99% confidence intervals, respectively.

Overall, the results are in line with our hypotheses in that trajectories characterised by stable marital unions (cluster 1) were associated with higher subjective health and higher life satisfaction for men and women (hypothesis 1a). Adults whose trajectories were characterised by singlehood (5) and divorce (3), in turn, experienced the lowest well-being in old age (hypothesis 1b). Our results also highlight that remarried older adults (2) did not display substantially lower levels of well-being than those in their first marital unions. More than one marriages were associated with lower assessments of subjective health only among women, but differences in effect sizes were modest. Finally, trajectories characterised by serial cohabitation (4) were associated with lower levels of well-being. These patterns remained after exogenous controls (model 2) and even in the over-controlled model (model 3). (For full models, see Tables 4, 5, 6, and 7)

Overall, the general pattern was similar for both sexes, although the association between singlehood and life satisfaction appears stronger for men. Robustness checks with alternative well-being measures suggested that the associations seemed to hold more consistently for men (hypothesis 1c). Findings are described in greater detail below and in Fig. 10).

**Fig. 3:**
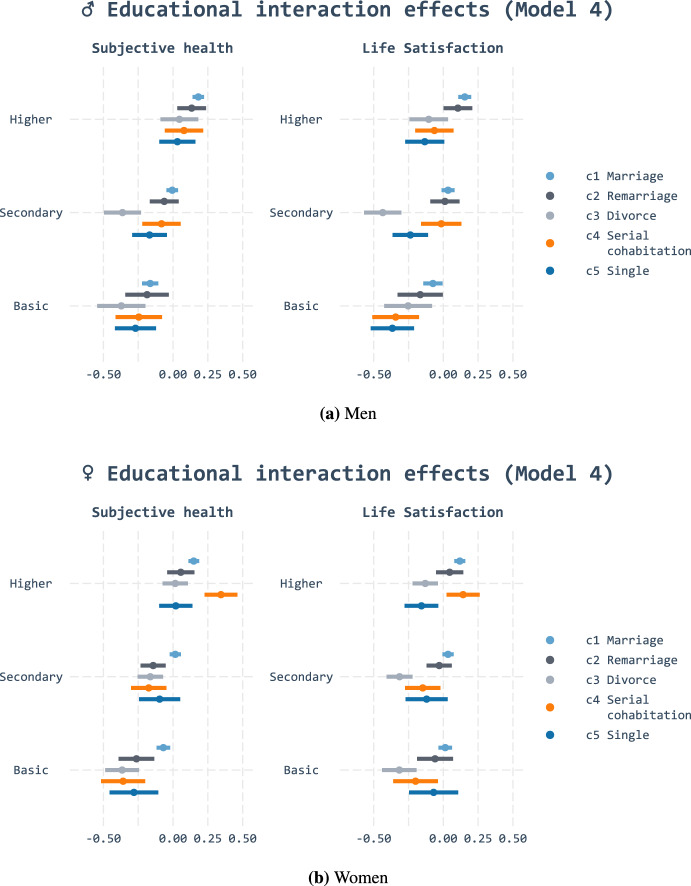
Predicted values of linear regression for subjective health and life satisfaction with educational interaction effects (Model 4)

**Fig. 4:**
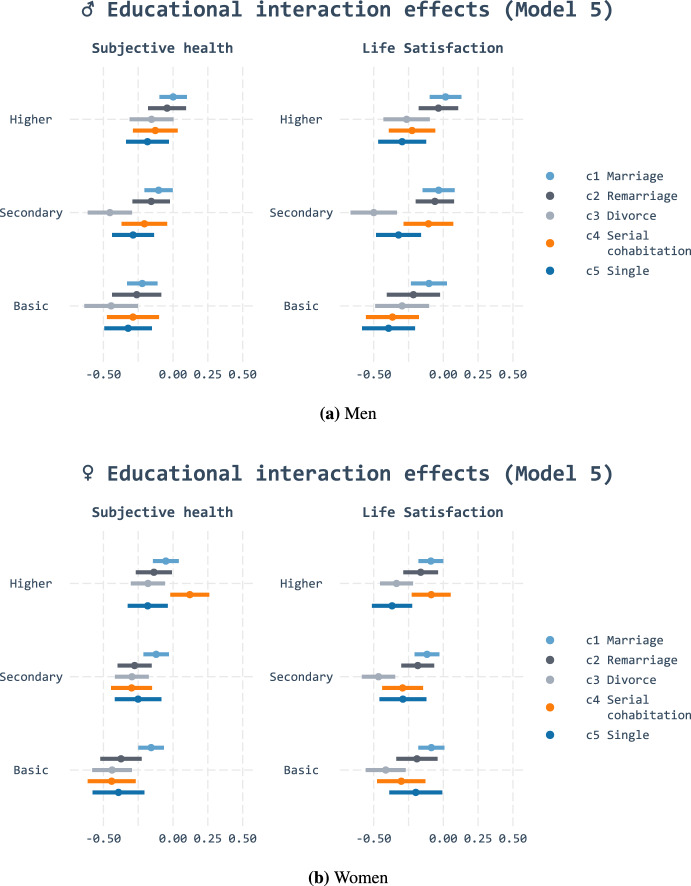
Predicted values of linear regression for subjective health and life satisfaction with educational interaction effects (Model 5)

**Fig. 5:**
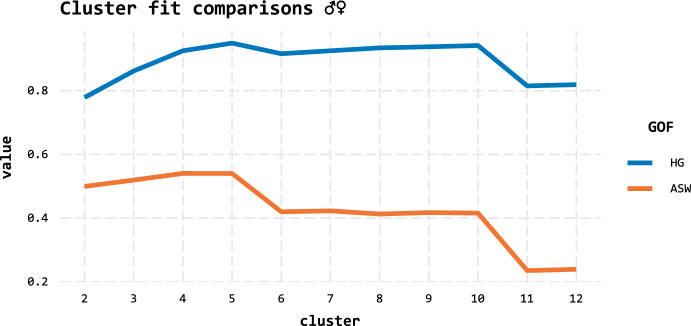
The goodness-of-fit tests for the number of clusters

**Fig. 6:**
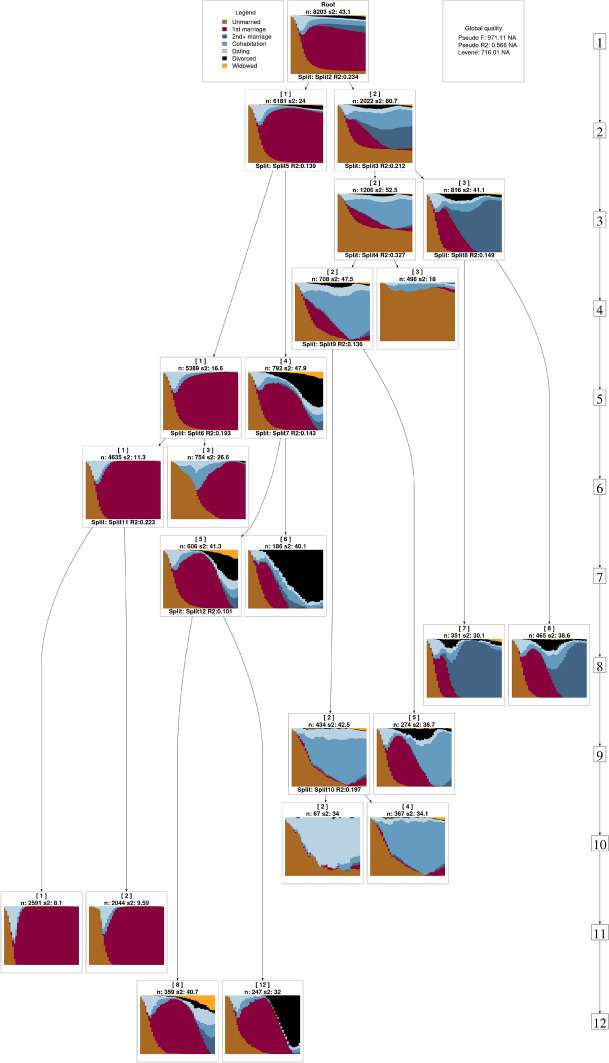
Sequence regression tree displaying all options for the number of clusters

**Fig. 7:**
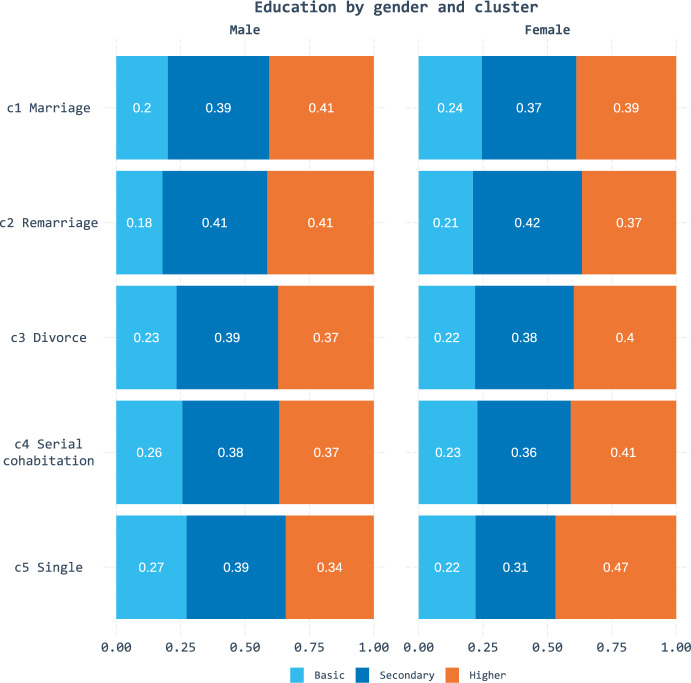
Educational attainment by sex and partnership trajectory

**Fig. 8:**
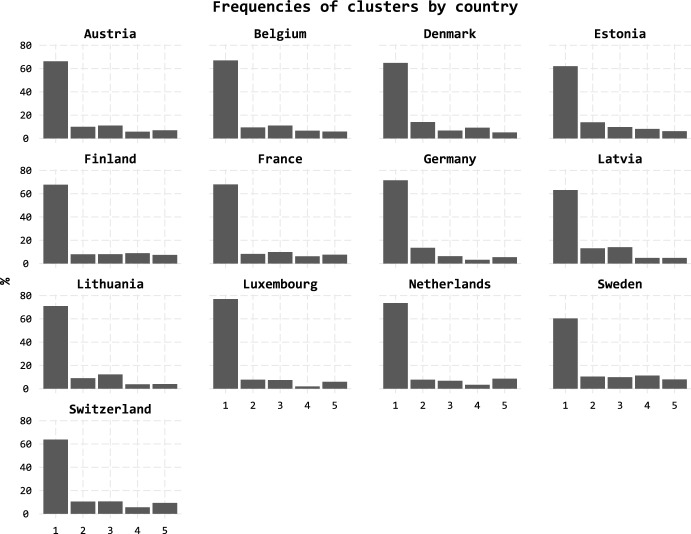
Cluster membership percentages by country

**Fig. 9:**
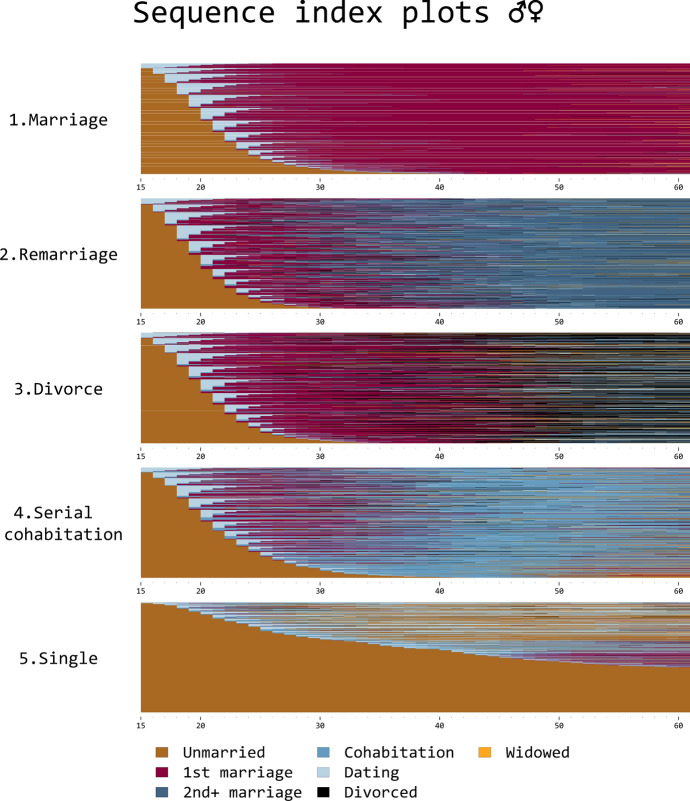
Sequence index plots for partnership history clusters for Western and Northern European men and women born in 1945–1957 at the age of 15–60

Finally, we assess whether the association between well-being and partnership is contingent on education. Here, we contrast predictions based on resource substitution and multiplication: we ask whether education amplifies advantages attached to histories with one stable marriage or, conversely, whether stable marital unions mitigate the lower well-being of low-educated individuals. Figures 3, 4, and 11 display the *predicted values* of all education and cluster combinations by sex conditional on the respective controls.

The results show that the negative association between the divorce cluster and subjective health was stronger among the basic and secondary educated. Among women, we find similar interaction effect for life satisfaction, albeit not as strong. This means that the gap in well-being between those in their first marital union and those who did not remarry after divorce is larger among low-educated individuals than highly educated individuals. These trends were most prominent in model 4, which controlled for age. The patterns remained throughout models 5 and 6, although not as clearly. We believe that this is mainly due to sample size reduction: it is not so much the smaller difference in point estimates but the wider confidence intervals that seem to dispel the patterns as more controls are introduced. This should not surprise us: as Gelman (2018) has demonstrated, one would need as much as 16 times more data to estimate interaction effects than the main effect.

These results point to an educational gradient in line with resource substitution (hypothesis 2b). Those who do not have the resources attached to higher education appear to be more reliant on the resources that years in marriages can provide: trajectories characterised by divorce without remarriage result in pronounced negative well-being associations for those with less education. High education, in turn, did not predict additional well-being for those with stable marital histories. Our results do not accordingly support resource multiplication (hypothesis 2a). This also indicates that stable marriages predict health and quality of life similarly for all educational groups.

The findings point out that patterns are more complex than theoretically assumed. In contrast to divorce, the negative associations of singlehood are not more pronounced among those with basic or secondary education. Although the singlehood cluster (5) had the lowest well-being and stable marriages (1) the highest, the educational gradient was constant within both clusters. The educational gradient also seems to vary by sex: while serial cohabitation (4) appeared to be disadvantageous for all men, the association varied among women by education. Highly educated women with serial cohabitation fared best out of all groups. Those with basic or secondary education experienced equally negative well-being following their complex partnership histories as those in the permanent singlehood (5) cluster.

### Robustness Checks

**Fig. 10:**
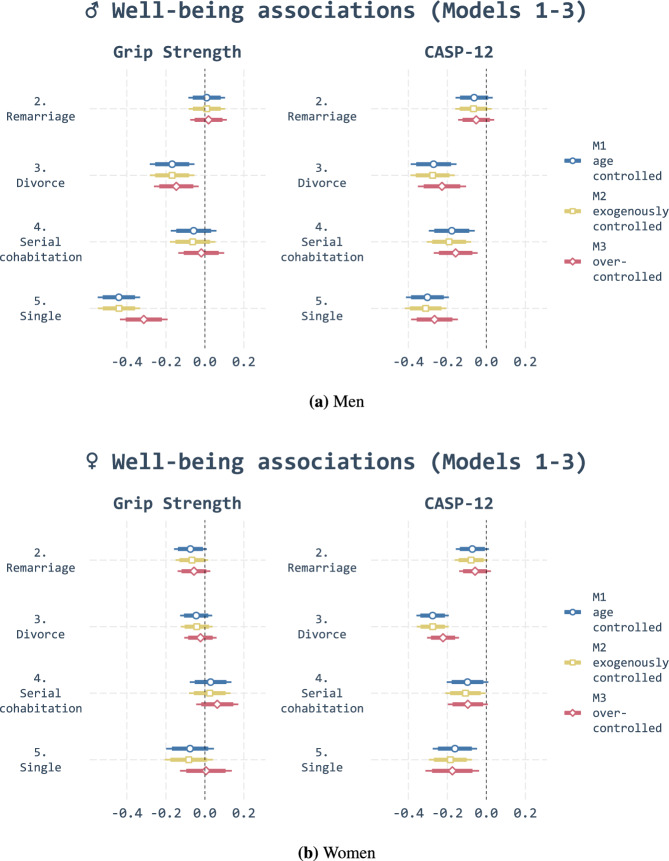
Linear regression for grip strength and CASP-12 without interaction effects (Models 1,2 & 3). *Note:* Marriage (c1) is the reference category. *Note:* Marriage (c1) is the reference category

**Fig. 11:**
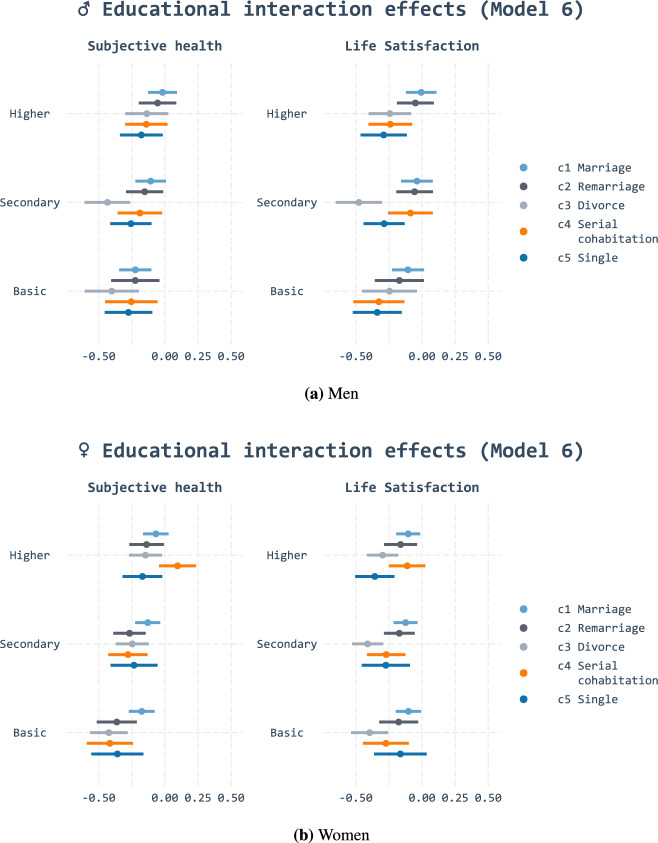
Predicted values of linear regression for subjective health and life satisfaction with educational interaction effects (Model 6)

**Fig. 12:**
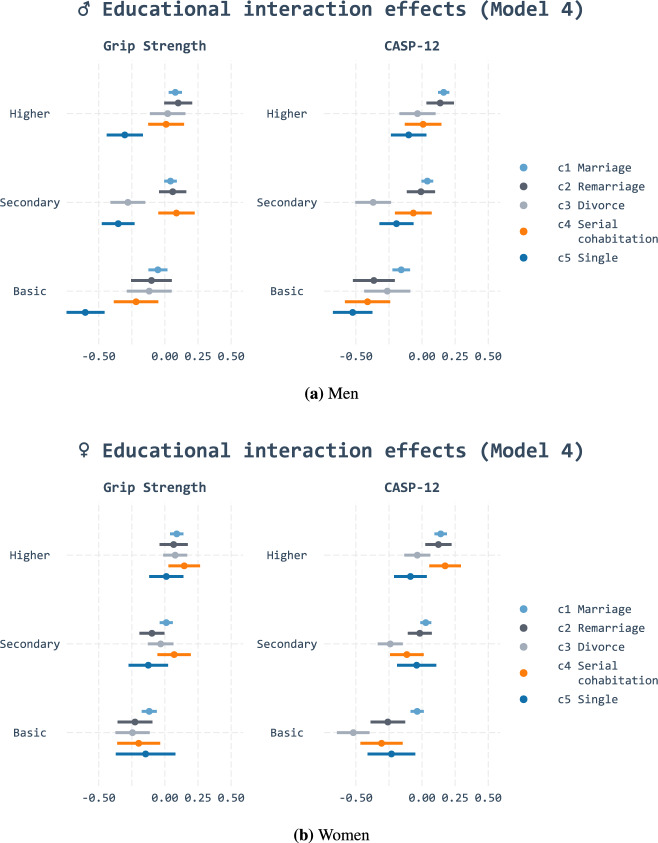
Predicted values of linear regression for grip strength and CASP-12 with educational interaction effects (Model 4)

The results were similar when measured with grip strength and CASP-12 (Figs. 10, 12). Grip strength deviated from other response variables: effect sizes were larger for men and smaller for women, for whom the effect sizes were almost zero. The direction of the association was mostly as expected. Still, the near disappearance of association in grip strength implies that the relationship between partnership and well-being, or health, is more robust for men. Grip strength for men in the cluster characterised by singlehood was the strongest negative association that we found in this study: around 0.4 standard deviations lower score than men in the stable first marriage cluster. Those with basic education had even lower scores for both for grip strength and CASP-12. Other than those, we did not find support for interaction effects or resource substitution among those in the single cluster.

As subjective health and life satisfaction were measured on 5- and 11-point scales, we also estimated each model using ordinal regression. The results were in line with the OLS (results omitted for brevity).

**Fig. 13:**
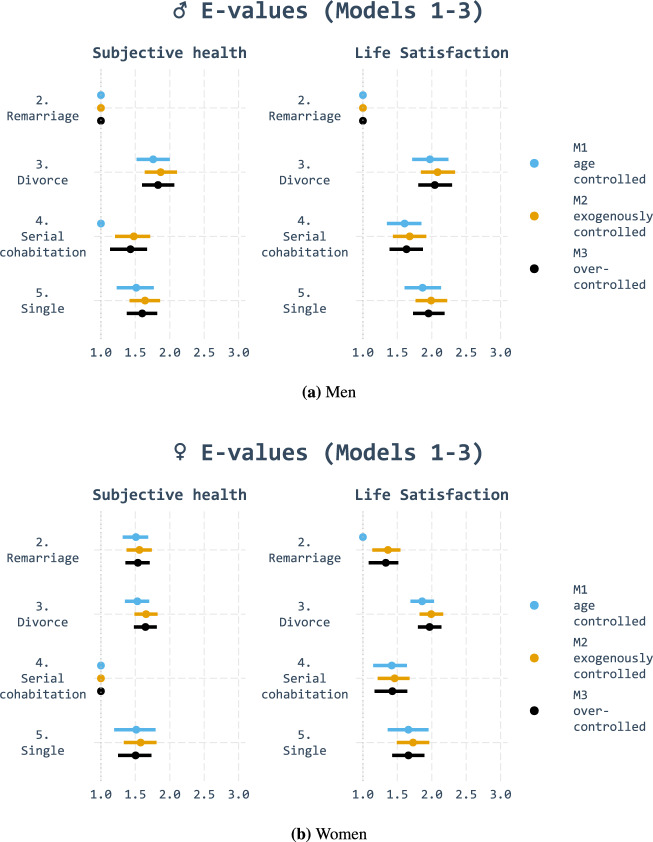
E-values to estimate the magnitude that an unmeasured confounder would have to have in order to explain away the association. *Note:* Marriage (c1) is the reference category

Estimating how an unobserved confounder would change the associations further corroborated our findings. The E-values in Fig. 13 are mainly around 1.5 and 2.3, meaning that, on the risk ratio scale, an unmeasured confounder associated both with partnership histories and later life outcomes would have to have a 1.5 to 2.0-fold effect in order to explain away the association. The remarriage (2) cluster among men and serial cohabitation (4) cluster among women were exceptions: even a small confounder would have been enough to nullify the findings.

**Fig. 14:**
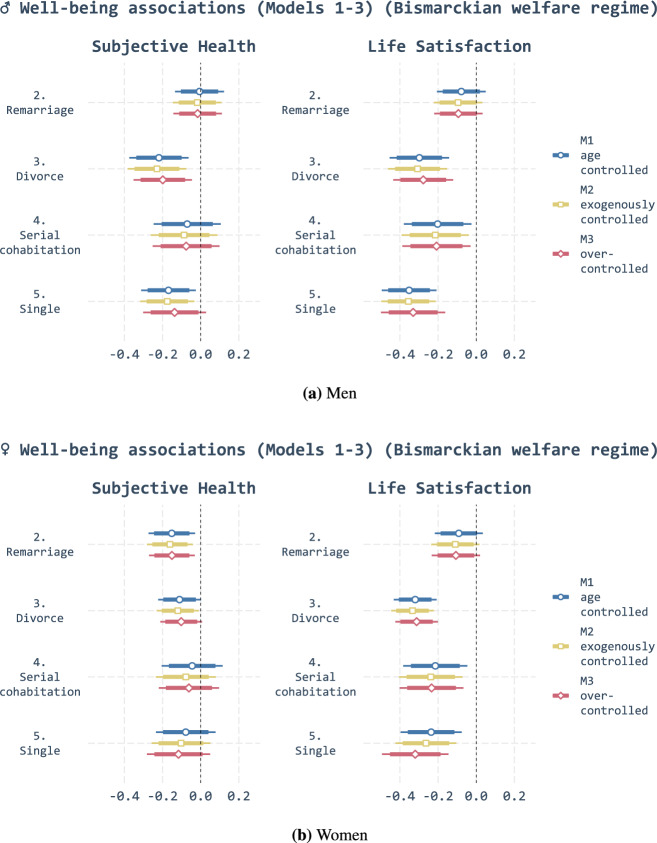
Linear regression for subjective health and life satisfaction without interaction effects (Models 1,2 & 3)—Bismarckian welfare regime. *Note:* Marriage (c1) is the reference category

**Fig. 19:**
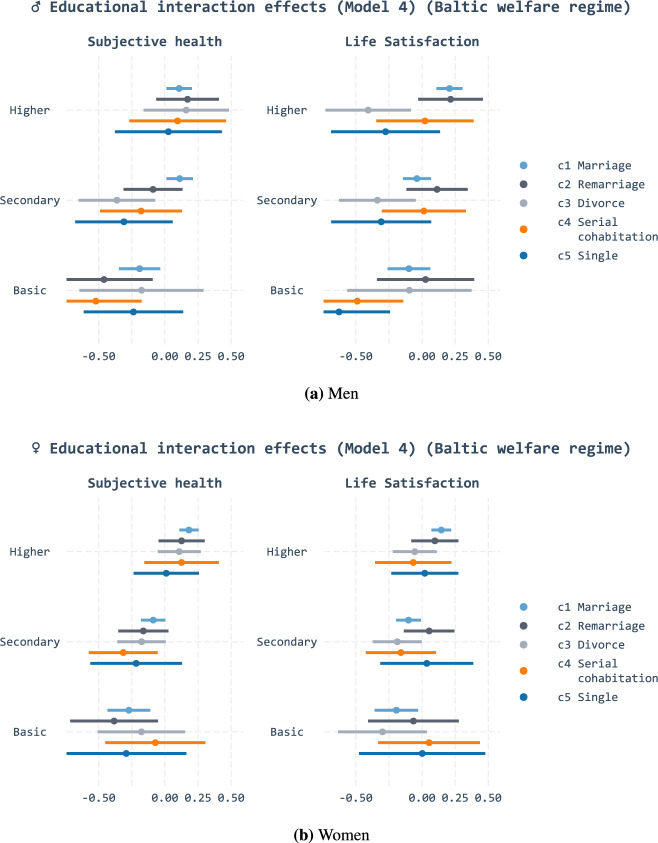
Predicted values of linear regression for subjective health and life satisfaction with educational interaction effects (Model 4)—Baltic welfare regime

The welfare regime-specific analyses tell essentially the same story as the aggregated one, but there are some nuances: highly educated women in the serial cohabitation cluster (4) in the Baltic welfare regime did not display any positive well-being interaction effects. The trend was strongest in Scandinavian countries, but distinguishable in the Bismarckian welfare regime as well. In addition, the well-being associations of the remarriage cluster (2) were somewhat inconsistent in the region-specific analyses, which is probably best explained by small effect sizes and decreased sample sizes (see Fig. 14 through Fig. 19). It is also interesting that the nordic men in the divorce (3) cluster had relatively much lower well-being than men in the other welfare regimes.

In order to check that the results were consistent across our study cohorts, we also split the analysis for respondents born between 1945 and 1950 and 1951 and 1957. The results between the two age groups resembled one another. The only notable difference was that the those who belonged to the divorce cluster in the younger cohorts, had slightly lower well-being compared with those in the marriage cluster within respective cohorts, regardless which measure of well-being we used. Also men in the older cohort who belonged to the single cluster had lower grip strength in comparison with the marriage cluster, than their younger counterparts (results available upon request).

**Fig. 20:**
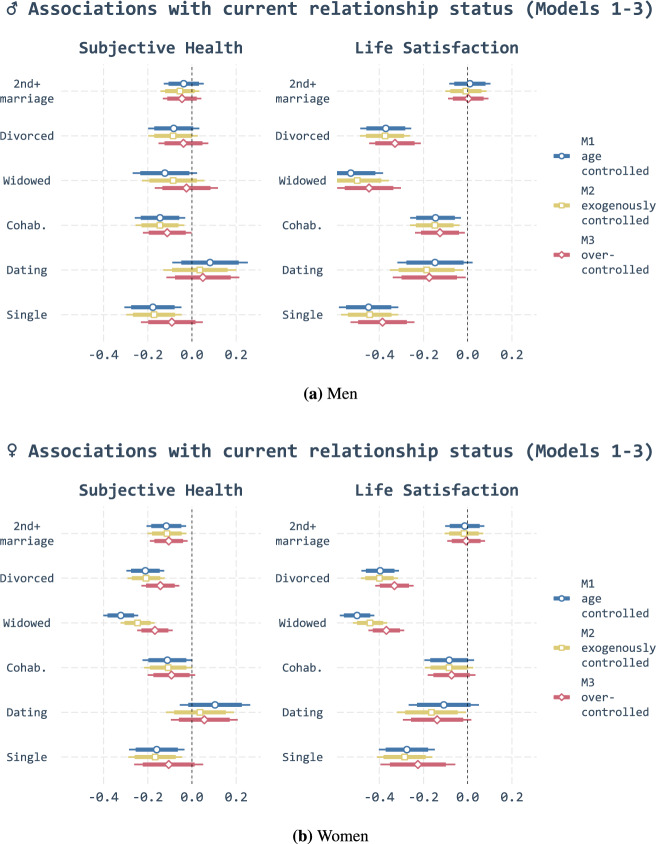
Linear regression with current relationship status for subjective health and life satisfaction without interaction effects (Models 1,2 & 3). *Note:* Marriage (c1) is the reference category

**Fig. 21:**
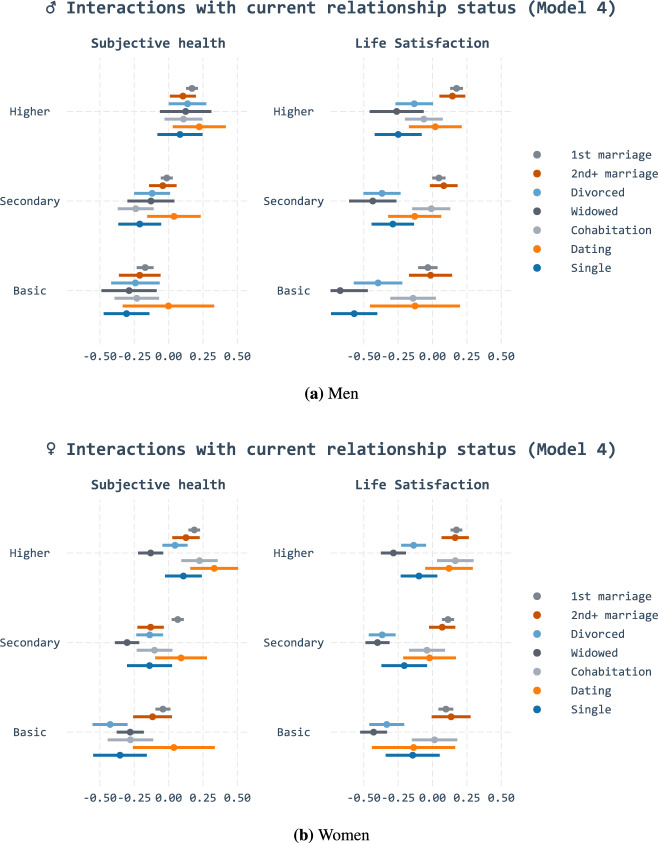
Linear regression with current relationship status for subjective health and life satisfaction with educational interaction effects (Model 4)

When the analyses were run with current relationship status instead of cluster membership, the associations were almost identical. Men in the divorce cluster had around 0.2 standard deviations lower subjective health compared to men in marriage cluster than did men whose current status was divorced compared to those who were currently married. Within the serial cohabitation cluster, those who were dating generally had better well-being than the ones that cohabited (Figs. 20, 21). The widowed and divorced had similar well-being.

In sum, the results were mainly corroborated with alternative response variables; ordinal regression produced similar results; the results were similar across different welfare regimes; sensitivity analysis revealed that the associations were rather resistant to potential unmeasured confounding, especially in clusters with extensive periods without a partner; stratifying the analyses by cohort groups led to similar outcomes, and exchanging cluster membership for current relationship introduced only nuances to the bigger picture.

## Discussion and Conclusion

We investigated, for the first time, how partnership histories are linked with health and quality of life in old age by educational groups. Our findings show that stable marital histories are steadily associated with well-being in old age across all educational groups as well as across different welfare regimes in Northern and Western Europe. Interaction analyses further indicate that lower education together with trajectories characterised by divorce without repartnering predict pronounced adverse well-being in old age, especially among men.

Romantic relationships seem to matter for individuals’ well-being throughout the whole life course, which supports the theories of cumulative advantage and disadvantage. The general trend was that the less attached individuals were from one stable marital union, the less happy and healthy they were. The findings also provided a more nuanced view on those in partnerships: individuals with stable second or higher-order marriages did not consistently have lower well-being. This finding differs from previous research (Zahl-Olsen et al., 2019; Booth & Edwards, 1992) that provided a starker difference between trajectories characterised by remarriages and those of first marriages.

As opposed to earlier literature (Zimmermann & Hameister, 2019; Jung, 2023), we did not find substantial differences between analyses conducted with relationship history clusters or current relationship status. One the one hand, our results suggest that when relationship history information is not available, current status is a decent proxy for relationship history. The current status often reflects the life course, especially for stable first marriages and lifelong singlehood. On the other hand, we also found some nuances using the trajectory typology approach. Those who were currently dating had similar well-being with those who were currently married. But when looking at the trajectories where dating is commonplace, the well-being is similar to those who are unpartnered. Similarly, presenting trajectories show long-term complexities: the fact that cohabitational relationships tend not to be as long lasting as marital unions serves as a reasonable explanation as to why life courses characterised by cohabitation do not come with similar well-being associations as those characterised by marriage. In addition, men in a cluster characterised by divorce had clearly lower subjective health than men whose current status was divorced, which could also indicate that studying trajectories gives some additional information than just studying the current state.

One substantial contribution of the paper was its differentiation between resource multiplication and substitution. Our results suggest that those with fewer resources might have inferior opportunities to compensate for life course events that potentially impede well-being. Conversely, the highly educated could also be less vulnerable to unstable partnership trajectories (Mirowsky & Ross, 2005). Evidence for resource substitution was stronger among men, which could indicate that men are more reliant on spousal support than women in later life (Ajrouch et al., 2005).

Our results also pointed to patterns that did not follow from the chosen theoretical framework: highly educated women, whose relationship trajectories were characterised by repeated cohabiting unions, experienced higher levels of well-being than those in first marriage cluster. It is possible that women in our study cohort who moved from one cohabitation spell to another were a distinct group with resources and characteristics different from the general population. Arriving at this kind of trajectory might also have been more of a conscious choice than for others in the cluster. As associations did not disappear after controls, it is possible that the mechanisms between serial cohabitation and well-being could be different for highly educated women, especially in the Scandinavian countries, as demonstrated by our robustness checks.

A pseudo-panel has the unfortunate feature of excluding the most vulnerable segments of population. Not only are those who are better off and in relationships more likely to participate, but those who have passed away are excluded by design. As a result, the resource substitution that we observed here are most likely to be stronger in reality (cf. axiom 5 in Ferraro et al., 2009). Although we did measure the effect of an unmeasured confounder, it would have been easier to identify any confounders if the panel was not retrospective. Unmeasured traits could have included, for instance, relationship quality, personal characteristics, contentment with relations with own children and near kin, and mutual friends. A recent study using SHARE data from Finland found that early and stable marital unions had higher relationship quality, suggesting a proximate mechanism for the beneficial associations between partnership history and well-being reported here (Tambellini et al., 2024).

We chose to aggregate our analysis for Western and Northern Europe as we were interested in the general associations of union histories as opposed to country-specific variations. The current literature does not support the hypothesis that the contribution of relationship status (or histories) would have notable cross-national variation (Diener et al., 2000; Verbakel, 2012). Empirically, we found no distinct patterns in the country distributions by the clusters, and the well-being associations were similar across the Scandinavian, Bismarckian and Baltic welfare regimes. It is still possible that some intriguing between-country variations were lost in the analysis. Likewise, had we included also Southern and Eastern European countries, we would probably have observed more traditional relationship histories (Perelli-Harris & Amos, 2015), but presumably similar well-being associations (cf. Diener et al. 2000; Verbakel 2012).

Future research could attempt to replicate whether the results hold for younger cohorts. An interesting question is whether the associations are chiefly due to the benefits of the stable marital histories themselves, or whether social life, sanctions, and internalised roles will play out differently for the cohorts that have entered the marriage market in the 21st century, and if that will have changes in the well-being associations of stable long marriages. On the one hand, both selection into unions and inequalities in health have increased with education over the past decades (Meara et al., 2008; Corti & Scherer, 2021), which would predict the increase of the well-being differences in the future. On the other hand, family life patterns and ideals have transformed since the mid and late 20th century (Lesthaeghe & van de Kaa, 1986). Hence, leading a non-traditional family life course might not have the same social implications as before, and the differences in well-being outcomes could converge.

In addition to studying younger cohorts, qualitative inquiries could further investigate the mechanisms behind the associations, such as the role of children, social networks, or financial support. Register studies could dig deeper into the combined effect of education, the number of children, and other demographic characteristics, and shed more light on the causal mechanisms beyond what we were able to capture here with the unmeasured confounder analysis. More generally, a larger sample size would be ideal for models including interaction effects (Gelman, 2018).

We conclude that life courses characterised by stable marriages tend to be coupled with good health and high quality of life, unstable and single histories less so. Low educational attainment together with partnership trajectories characterised by divorce have pronounced lower negative well-being associations. Our results hint at family formation patterns that may foster well-being and mechanisms that potentially boost or buffer the outcomes. Our study also helps policymakers identify vulnerable subgroups and highlight the persistent link between romantic relationships and healthy, happy ageing.

## Figures and Tables

**Fig. 15:**
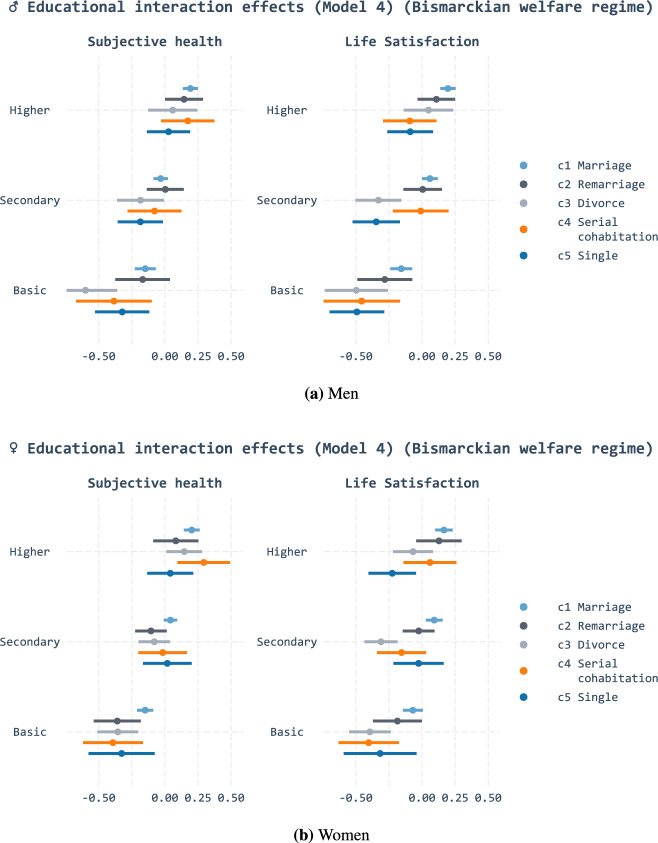
Predicted values of linear regression for subjective health and life satisfaction with educational interaction effects (Model 4)—Bismarckian welfare regime

**Fig. 16:**
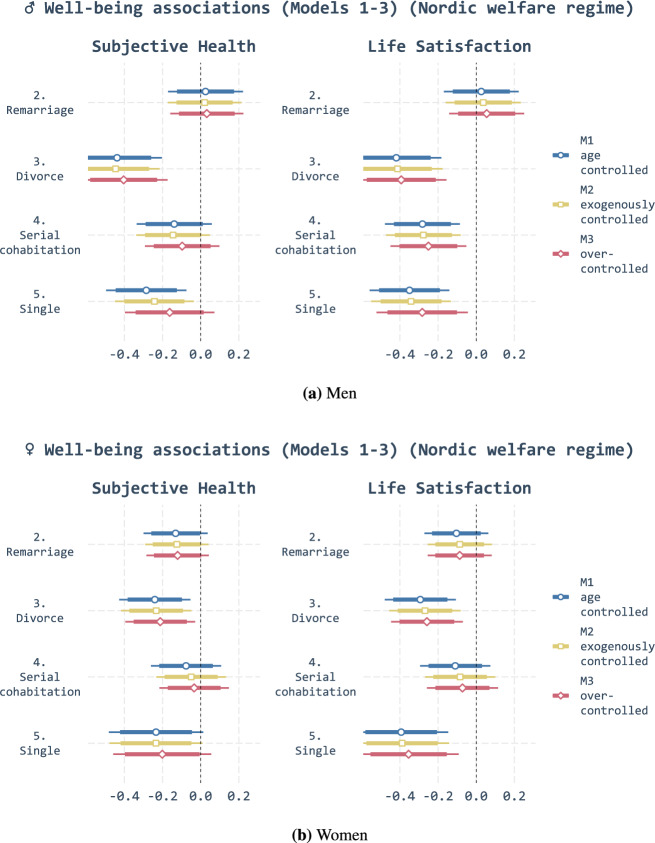
Linear regression for subjective health and life satisfaction without interaction effects (Models 1,2 & 3)—Nordic welfare regime. *Note:* Marriage (c1) is the reference category

**Fig. 17:**
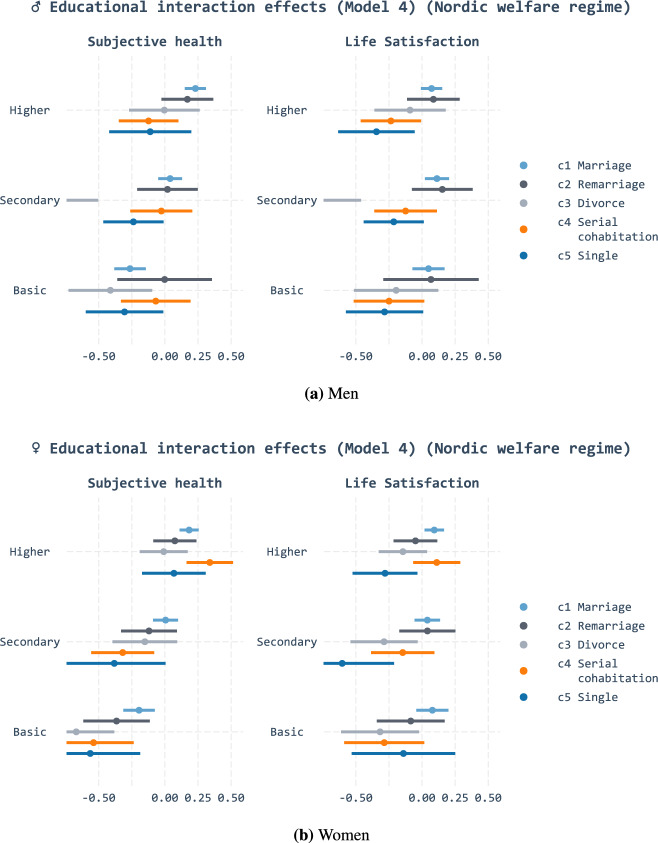
Predicted values of linear regression for subjective health and life satisfaction with educational interaction effects (Model 4) - Nordic welfare regime

**Fig. 18:**
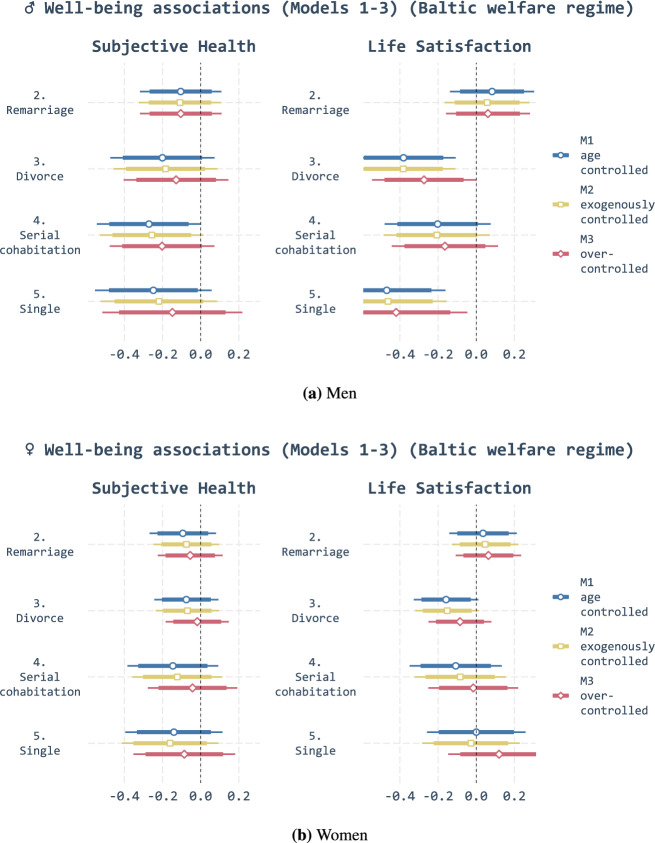
Linear regression for subjective health and life satisfaction without interaction effects (Models 1,2 & 3)—Baltic welfare regime. *Note:* Marriage (c1) is the reference category

## Data Availability

The data used in this article are available free-of-charge for registered researchers via the SHARE project website: https://share-eric.eu/data/data-access.

## Appendix

See Figs. 5, 6, 7, 8, 9, 10, 11, 12, 13, 14, 15, 16, 17, 18, 19, 20 and 21

See Tables 3

**Table 3:** Multinomial regression: relative risk ratios for belonging to a cluster

	Cluster			
	2	3	4	5
	(1)	(2)	(3)	(4)
Belgium	0.937	0.992	1.145	0.829
	(0.113)	(0.107)	(0.139)	(0.134)
Denmark	1.443$$^{***}$$	0.627$$^{***}$$	1.639$$^{***}$$	0.749$$^{*}$$
	(0.114)	(0.130)	(0.142)	(0.152)
Estonia	1.480$$^{***}$$	0.949	1.513$$^{***}$$	0.943
	(0.110)	(0.113)	(0.139)	(0.139)
Finland	0.779	0.713$$^{**}$$	1.521$$^{**}$$	1.027
	(0.153)	(0.150)	(0.164)	(0.165)
France	0.813$^{*}$	0.876	1.057	1.065
	(0.122)	(0.115)	(0.148)	(0.135)
Germany	1.255$$^{**}$$	0.534$$^{***}$$	0.516$$^{***}$$	0.728$$^{**}$$
	(0.111)	(0.127)	(0.173)	(0.144)
Latvia	1.381$$^{**}$$	1.336$$^{**}$$	0.884	0.723
	(0.149)	(0.144)	(0.218)	(0.213)
Lithuania	0.849	1.038	0.606$$^{**}$$	0.532$$^{***}$$
	(0.156)	(0.141)	(0.224)	(0.214)
Luxembourg	0.667$$^{**}$$	0.577$$^{***}$$	0.289$$^{***}$$	0.730
	(0.175)	(0.176)	(0.313)	(0.199)
Netherlands	0.700$$^{**}$$	0.558$$^{***}$$	0.517$$^{***}$$	1.110
	(0.155)	(0.159)	(0.221)	(0.159)
Sweden	1.151	0.986	2.155$$^{***}$$	1.251
	(0.120)	(0.119)	(0.138)	(0.138)
Switzerland	1.095	1.003	1.012	1.392$$^{**}$$
	(0.128)	(0.125)	(0.167)	(0.141)
Constant	0.150$$^{***}$$	0.166$$^{***}$$	0.087$$^{***}$$	0.106$$^{***}$$
	(0.088)	(0.084)	(0.112)	(0.103)
Akaike Inf. Crit	39,401.340	39,401.340	39,401.340	39,401.340

*Note:* $$^{*}$$p<0.1; $$^{**}$$p<0.05; $$^{***}$$p<0.01

Austria is the reference country

, 4

**Table 4:** Linear regression for subjective health (men)

	Model 1	Model 2	Model 3
(Intercept)	$$0.66^{***}$$	0.02	0.15
	(0.20)	(0.19)	(0.21)
2.Remarriage	$-0.04$	$-0.05$	$-0.03$
	(0.04)	(0.04)	(0.04)
3.Divorce	$$-0.25^{***}$$	$$-0.26^{***}$$	$$-0.21^{***}$$
	(0.04)	(0.04)	(0.04)
4.Serial cohabitation	$$-0.10^{*}$$	$$-0.12^{**}$$	$$-0.08$$
	(0.05)	(0.04)	(0.04)
5.Single	$$-0.17^{***}$$	$$-0.17^{***}$$	$$-0.13^{**}$$
	(0.04)	(0.04)	(0.05)
Age	$$-0.01^{**}$$	0.00	$$-0.00$$
	(0.00)	(0.00)	(0.00)
Experienced hunger as a minor		$-0.16$	$-0.16$
		(0.09)	(0.09)
Features at childhood home		$$0.21^{***}$$	$$0.15^{***}$$
		(0.01)	(0.03)
Books at childhood home		0.02	0.00
		(0.01)	(0.01)
Rooms at childhood home		$$0.08^{**}$$	$$0.07^{**}$$
		(0.02)	(0.02)
Not living with both biological parents when ten		$$-0.13^{***}$$	$$-0.11^{**}$$
		(0.03)	(0.03)
1 child (ref 2+)			$-0.05$
			(0.03)
0 children (ref 2+)			$-0.04$
			(0.04)
Secondary education (ref Tertiary)			$$-0.10^{***}$$
			(0.03)
Basic education (ref Tertiary)			$$-0.19^{***}$$
			(0.03)
Log income			0.13
			(0.06)
nimp	6	6	6
nobs	8203	8203	8203
$\hbox {R}^2$	0.01	0.07	0.10
Adj. $\hbox {R}^2$	0.01	0.07	0.10

$$^{***}p<0.001$$; $$^{**}p<0.01$$; $$^{*}p<0.05$$

, 5

**Table 5:** Linear regression for life satisfaction (men)

	Model 1	Model 2	Model 3
(Intercept)	$$-0.31$$	$$-0.83^{***}$$	$$-0.76^{***}$$
	(0.21)	(0.21)	(0.20)
2.Remarriage	$-0.04$	$-0.05$	$-0.04$
	(0.04)	(0.04)	(0.04)
3.Divorce	$$-0.33^{***}$$	$$-0.34^{***}$$	$$-0.29^{***}$$
	(0.05)	(0.04)	(0.05)
4.Serial cohabitation	$$-0.18^{***}$$	$$-0.19^{***}$$	$$-0.16^{***}$$
	(0.05)	(0.04)	(0.04)
5.Single	$$-0.30^{***}$$	$$-0.30^{***}$$	$$-0.25^{***}$$
	(0.04)	(0.04)	(0.05)
Age	0.01	$$0.01^{***}$$	$$0.01^{***}$$
	(0.00)	(0.00)	(0.00)
Experienced hunger as a minor		$-0.12$	$-0.11$
		(0.10)	(0.10)
Features at childhood home		$$0.18^{***}$$	$$0.13^{***}$$
		(0.01)	(0.03)
Books at childhood home		0.02	0.01
		(0.01)	(0.01)
Rooms at childhood home		$0.04^{*}$	0.02
		(0.02)	(0.01)
Not living with both biological parents when ten		$$-0.09^{**}$$	$$-0.07^{*}$$
		(0.04)	(0.03)
1 child (ref 2+)			$-0.07^{*}$
			(0.03)
0 children (ref 2+)			$-0.05$
			(0.04)
Secondary education (ref Tertiary)			$-0.03$
			(0.03)
Basic education (ref Tertiary)			$$-0.09^{**}$$
			(0.03)
Log income			0.16
			(0.07)
nimp	6	6	6
nobs	8203	8203	8203
$\hbox {R}^2$	0.01	0.06	0.09
Adj. $\hbox {R}^2$	0.01	0.06	0.09

$$^{***}p<0.001$$; $$^{**}p<0.01$$; $$^{*}p<0.05$$

, 6

**Table 6:** Linear regression for subjective health (women)

	Model 1	Model 2	Model 3
(Intercept)	$$1.32^{***}$$	$$0.50^{**}$$	$$0.57^{**}$$
	(0.18)	(0.18)	(0.17)
2.Remarriage	$$-0.14^{***}$$	$$-0.14^{***}$$	$$-0.12^{***}$$
	(0.03)	(0.03)	(0.03)
3.Divorce	$$-0.18^{***}$$	$$-0.18^{***}$$	$$-0.13^{***}$$
	(0.03)	(0.03)	(0.03)
4.Serial cohabitation	$-0.05$	$-0.06$	$-0.04$
	(0.04)	(0.04)	(0.04)
5.Single	$$-0.13^{**}$$	$$-0.15^{***}$$	$$-0.13^{*}$$
	(0.04)	(0.04)	(0.05)
Age	$$-0.02^{***}$$	$$-0.01^{*}$$	$$-0.01^{*}$$
	(0.00)	(0.00)	(0.00)
Experienced hunger as a minor		$$-0.22^{**}$$	$$-0.20^{*}$$
		(0.08)	(0.08)
Features at childhood home		$$0.19^{***}$$	$$0.12^{***}$$
		(0.02)	(0.02)
Books at childhood home		$$0.05^{***}$$	$$0.03^{*}$$
		(0.01)	(0.01)
Rooms at childhood home		0.10	0.07
		(0.05)	(0.03)
Not living with both biological parents when ten		$$-0.14^{***}$$	$$-0.12^{***}$$
		(0.03)	(0.03)
1 child (ref 2+)			$$-0.10^{***}$$
			(0.03)
0 children (ref 2+)			$-0.02$
			(0.04)
Secondary education (ref Tertiary)			$$-0.09^{***}$$
			(0.02)
Basic education (ref Tertiary)			$$-0.17^{***}$$
			(0.03)
Log income			$$0.20^{***}$$
			(0.03)
nimp	6	6	6
nobs	10007	10007	10007
$\hbox {R}^2$	0.01	0.09	0.13
Adj. $\hbox {R}^2$	0.01	0.09	0.13

$$^{***}p<0.001$$; $$^{**}p<0.01$$; $$^{*}p<0.05$$

and 7

**Table 7:** Linear regression for life satisfaction (women)

	Model 1	Model 2	Model 3
(Intercept)	0.15	$$-0.50^{**}$$	$$-0.46^{*}$$
	(0.18)	(0.18)	(0.18)
2.Remarriage	$-0.07^{*}$	$-0.08^{*}$	$-0.06$
	(0.03)	(0.03)	(0.03)
3.Divorce	$$-0.30^{***}$$	$$-0.30^{***}$$	$$-0.25^{***}$$
	(0.03)	(0.03)	(0.03)
4.Serial cohabitation	$$-0.10^{*}$$	$$-0.11^{**}$$	$$-0.09^{*}$$
	(0.04)	(0.04)	(0.04)
5.Single	$$-0.19^{***}$$	$$-0.21^{***}$$	$$-0.18^{**}$$
	(0.04)	(0.04)	(0.05)
Age	$$-0.00$$	$$0.01^{**}$$	$$0.01^{**}$$
	(0.00)	(0.00)	(0.00)
Experienced hunger as a minor		$$-0.29^{***}$$	$$-0.27^{***}$$
		(0.08)	(0.08)
Features at childhood home		$$0.17^{***}$$	$$0.10^{***}$$
		(0.01)	(0.02)
Books at childhood home		$$0.04^{**}$$	$$0.03^{**}$$
		(0.01)	(0.01)
Rooms at childhood home		0.04	0.01
		(0.03)	(0.02)
Not living with both biological parents when ten		$-0.07^{*}$	$-0.05$
		(0.03)	(0.03)
1 child (ref 2+)			$$-0.11^{***}$$
			(0.03)
0 children (ref 2+)			$-0.01$
			(0.04)
Secondary education (ref Tertiary)			$-0.03$
			(0.02)
Basic education (ref Tertiary)			$-0.01$
			(0.03)
Log income			$$0.21^{***}$$
			(0.03)
nimp	6	6	6
nobs	10007	10007	10007
$\hbox {R}^2$	0.01	0.06	0.10
Adj. $\hbox {R}^2$	0.01	0.06	0.10

$$^{***}p<0.001$$; $$^{**}p<0.01$$; $$^{*}p<0.05$$

## Footnotes

1 This paper uses data from SHARE Waves 1, 2, 3, 4, 5, 6, and 7 (DOIs: 10.6103/SHARE.w1.710, 10.6103/SHARE.w2.710, 10.6103/SHARE.w3.710, 10.6103/SHARE.w4.710, 10.6103/SHARE.w5.710, 10.6103/SHARE.w6.710, 10.6103/SHARE.w7.711). See Börsch-Supan et al. (2013) for methodological details.

2 We use the terms partnership histories and partnership trajectories interchangeably throughout this article.

3 Ages 15 to 60.

4 Singlehood, first marriages, higher-order marriages, dating, cohabitation, divorce, or widowhood.

5 "Would you say your health is... 1. Excellent, 2. Very good, 3. Good, 4. Fair, 5. Poor"

6 "On a scale from 0 to 10 where 0 means completely dissatisfied and 10 means completely satisfied, how satisfied are you with your life?"

7 The SHARE version of CASP-12 includes 12 statements on control, autonomy, pleasure, and self-realisation: My age prevents me from doing the things I would like to do; I feel that what happens to me is out of my control; I feel left out of things; I can do the things I want to do; Family responsibilities prevent me from doing the things I want to do; Shortage of money stops me from doing things I want to do; I look forward to each day; I feel that my life has meaning; On balance, I look back on my life with a sense of happiness; I feel full of energy these days; I feel that life is full of opportunities; I feel that the future looks good for me.
